# Transreal tracing: Queer-feminist speculations on disabled technologies

**DOI:** 10.1177/14647001221082299

**Published:** 2022-03-16

**Authors:** Katta Spiel

**Affiliations:** HCI Group, 27259 TU Wien, Austria

**Keywords:** Assistive technology, critical disability studies, feminist design, human–computer interaction, research through design, speculation

## Abstract

In a world where technologies often serve to amplify the persistent rendering of disability as an undesired deficit, what we need are empowering utopias concerning bodies, disabilities and assistive technologies. Specifically, I use Barad's article ‘Transmaterialities: Trans*/Matter/Realities and Queer Political Imaginings’ to illustrate how we might speculate on technologies that understand disabled bodies as affording potentials.

The Transreal Tracing Device reimagines our bodies as surfaces of possibility, encouraging explorations into how disabled bodies do and could look like. The speculative device offers an opportunity for positive renegotiations of disabled bodies as malleable and desirable – as ontologically indeterminate and transcendent.

In traversing theoretical approaches and using them to design queer-feminist utopias centring disabled people, the concept challenges dominant notions of disabilities and assistive technologies alike. I close by discussing implications for ability-based, participatory but even more so self-determined design, and how to shift the focus of disabled technologies towards potential, from support to appreciation, from isolation to kinship and, ultimately, from shame to pride.

## Cripping assumptions of assistive technologies

In technological design, disability and disabled people^1^ play a role almost exclusively within a context of assistive technologies. Framed as support devices, these are intended to ‘help’ disabled people to find workarounds in encountering an ableist environment. They embody a dominant framing of disability as an inherently individualised characteristic that is fundamentally undesirable (see: Wendell, 1996). Most of these technologies follow a medical model (Marks, 1997) instead of, for example, a social model (Oliver, 2013) in which environments are adapted to be inclusive (Hamraie, 2017) or even a mixed approach considering difference in embodiment (e.g. through a minority body as detailed by Barnes, 2016) that still requires accounting for (Shakespeare, 2013). In this tendency to normativise an expected non-disabled embodiment, assistive technologies largely comprise materialisations of what Campbell (2009) calls a *corporeal standard*. Hence, technologies for disabled people tend to articulate an *ableist* norm regarding which bodies are expected to interact with them, what is deemed useful and how people are supposed to live (see also: Gerling and Spiel, 2021). Bluntly put, these technologies are designed to assist in engaging with the world in ways that minimise both disabilities and their public presence, and they often do so by drawing on a rhetoric that hails the supremacy of independence. Though, ‘if our culture valued interdependence more highly, they [disabled people] could use that energy for more satisfying activities’ (Wendell, 1989: 118). Interdependence as a concept for disability studies has been articulated by Reindal (1999) among others to describe the lived experiences of disabled people who more pronouncedly rely on others to conduct their daily lives or achieve access to mainstream society. Following this understanding consequently means instead of seeing independent engagements with one's environments as the ultimately desirable goal that technologies could support (while actively ignoring the interdependencies on technologies this creates in return), technologies could value interdependence between humans and facilitate access intimacies^2^ instead of replacing human–human interactions. Doing so could provide us with the option to not only consider ability-based design (Wobbrock et al., 2011) but engage with disability cultures as a desirable starting point. Bennett et al. (2018) vividly illustrate how, for example, the notion of *interdependence* centres and more appropriately accounts for the lived experiences of disabled people in technology research, design and development.

Even when framing encounters in the context of participatory design, researchers often drive and gatekeep the purpose of technologies for disabled people, leading to a fundamental embedding of *othering as deviant* (see also: Spiel et al, 2019). Hence, these technologies fundamentally are not concerned with the needs, desires or requests of disabled people; they are shaped and constructed along ableist assumptions of what is deemed appropriate (Williams and Boyd, 2019), often oriented on the goal of understanding disabilities as deficits supposedly requiring cure and mitigation (Clare, 2017). Developments in this direction are prioritised in funding agencies, governmental initiatives or charities (cf. Morris, 2014). As these attempts are made within a framing of kindness, attention and care, it becomes difficult to address the underlying issues as ableist feelings are given more value than disabled people's survival.^3^ Subsequently, disability theories as well as political practices demand ‘rights not charities’ (Morris, 2014: 126). 

Strands of Human–Computer Interaction (HCI) research into the design of technologies for disabled people and particularly into ways of ensuring access to technological design (e.g. Hurst and Kane, 2013; Meissner et al., 2017) are somewhat attuned to the inherent necessity, and hence unique skillset, of disabled people to find strategies for being in a world fundamentally not made for them. Consequently, disabled scholars have called for an increase in the meaningful participation of disabled people in technology research about them (Mankoff et al., 2010; Spiel et al., 2020). Disabled people tend to have ample experiences in hacking devices, in shaping them for themselves, even early on as shown with research detailing how autistic youth, for example, use the game *Minecraft* in a way that could be understood as assistive (see: Ringland et al., 2016).^4^ This might partly be due to the experiences disabled people make in a world where most of the artefacts designed for them are designed by non-disabled people, leading to two fundamental problems of suitability. First, non-disabled people, by not sharing aspects of embodiment, have limited ways of knowing what entails a positive experience and, ultimately, a suitable device. ‘[I]t is implausible to think that non-disabled observers can understand the experience of disability by trying imaginatively to project themselves into the situation of people with disabilities’ (Mackenzie and Scully, 2007: 347). This first problem directly leads to the second: in trying to embody disabled experiences through empathy exercises, disabled people's lived experiences become overridden by non-disabled perspectives and interpretations (Bennett and Rosner, 2019). Similarly, disabled people's perspectives are epistemologically disregarded and academically excluded through a myriad of micro-aggressions (Brown and Leigh, 2018; Ymous et al., 2020).

My argument here is not that disabled people need to be given access to certain structures, but that our expertise and experiences, our designs and creations are devalued if they do not comply with the corporeal standards and the ableist notions upholding them. If perspectives stemming from non-disabled embodiment hold the power to define which technologies are appropriate for disabled people to engage with the world,^5^ they risk failing to recognise the expert strategies and situated knowledges of the very group they are trying to ‘help’. They risk overriding disabled people's desires with their own ideals about what constitutes satisfying living conditions.

[M]ost non-disabled people cannot wrap their minds around the possibility that someone can be disabled or ill and also work productively, have intimate relationships, or be happy. People without disabilities tend to assume that a person with a disability is unable to participate in most of the life activities they consider important. Thus, they infer that someone who can work at all cannot be significantly disabled. (Wendell, 1996: 4)

Hence, assistive technologies embody and articulate ableist aspects of corporeal standards, normative behaviour and expectations and embed them within a neoliberal, capitalist logic where human existences are valued according to how productive they can be in this system (Nishida, 2016). Deviations from these norms are categorised as matters in need of correction. ‘Technology comes into play […] in how performers have responded to the insistent presence of the medical gaze that pathologizes and infantilizes’ (Price, 2007: 82). With this pathologising comes an orientation towards mitigation or cure, which inherently assumes disability and sickness as undesirable and less than: ‘Most nondisabled people believe that I need to be repaired’ (Clare, 2017: 7). And yet, medical interventions and progress also build the foundations that extend the lives of many disabled people and the quality thereof (Clare, 2017). However, understanding disability and opportunities for technological innovation and development within this space solely from an angle of medicalisation leads to entire fields orienting themselves on how disabled people should change rather than identifying opportunities for self-determined interactions with technologies (see Spiel et al., 2019).

This analysis offers me an entry point to inquire into feminist perspectives on assistive technologies. I position my work within queer crip theories, and the overlap of queer theory and critical disability perspectives provides a fruitful point to go forward. Disabled people (by systematically being conceptualised as gendervoid, undesirable and without desire) inherently position themselves as queer simply by claiming gender and sexuality (McRuer, 2006; Kafer, 2013; Gallop, 2018). In the face of deeply ableist connotations of heteronormativity (McRuer, 2003), the very notion of disability as a multidimensional spectrum leads to a dissolution of supposedly static binaries of dis/abilities (Sandahl, 2003).

I use queer theory and *queering* as well as *cripping* to bring queer, crippled perspectives into the arena of assistive technologies. ‘Queering is problematizing apparently structural and foundational relationships with critical intent, and it may involve mischief and clowning as much as serious critique’ (Light, 2011: 432). My attempt to think about assistance and technology in modes of self-determination involves artful design explorations and meandering into silliness, but is grounded in passionate critique urging the fields to do better. In tracing perspectives specifically from feminist disability as well as materialist trans studies, I interrogate the notions of assistance and technology and embrace disabled experiences as not just ‘mere difference’ (Barnes, 2016) but as a source of expertise and strength without erasing difficulties or pain. The purpose of this article lies in speculating on unimaginable opportunities, to create fictional and surreal utopias that allow us to start dreaming about technologies for disabled people functioning beyond the narrow frame of assistance. We need to be able to imagine differences before we can start actualising them.

## Constructing theory through speculative design

My aim is to imagine designs of self-determined, positively encoded technologies for disabled people. Feminist studies of bodies, disabilities and assistive technologies predominantly concern themselves with the critique and analysis of a status quo, whereas design aims at future possibilities. Disability is, subsequently, of core interest to feminist theory. ‘Research through Design’ (Zimmerman et al., 2010) comprises a slowly establishing practice within technological research. The method allows for the investigation of theories and concepts by engaging directly with materials and concrete, practical constraints that emerge during creation. Simultaneously complementing and contrasting my approach, I argue for my work to be understood as constructing knowledge by exploring ‘theory through design’. Where research through design comprises a way to use design to think through theoretical concepts, my approach aims to identify theoretical contributions that designs can offer through themselves.

To this end, approaching the more speculative strand of design methodologies can illustrate a range of potential pathways for societies and a reflection on the direction that technological advancement is currently heading towards (Dunne and Raby, 2013). From a phenomenological perspective, speculating on feminist utopias for assistive technologies through design allows me to explore the resistance of designs as well as concepts and to encounter points of friction often removed from analytical inquiry in favour of a coherent and consistent narrative. Concretely, I engage with speculative notions of design by constructing what Dunne and Raby call ‘unrealities’. In that, I aim to ‘accept the fictional nature of design speculations and, rather than trying to convince the viewer that [my] ideas are “real,” learn to enjoy the unreality of speculation and the aesthetic opportunities it creates’ (Dunne and Raby, 2013: 134). Those opportunities are full of transformative potential. By imagining alternative aesthetics for disabilities, by venturing into the unreal, by engaging with potential and by doing so through creation, my speculations articulate the reclaiming of assistive technologies and technological design for disabled people more generally. ‘This sense of haptic engagement relates well to the (knowledge) politics of reclaiming the neglected: to speculative commitments that are about being in touch, relating with, and partaking in those worlds that are struggling to make their other visions not so much visible, but possible’ (Bellacasa, 2009: 311). In that regard, my work follows Price's call for thinking about disabilities within feminist paradigms, to ‘ask questions about the epistemology and ontology of disability as an embodied condition, personal and political’ (2007: 78). Doing so means embracing the ‘privilege of partial perspective’ (Haraway, 1988) to explore potential and possibility, to position alternatives.

I use speculative design as an approach to develop theory by decentring predominantly textual modes of theoretical production (following Derrida, [1976] 2016). Influenced by theoretical engagements from Feminist Disability Studies as well as Trans Studies, notably with the works of Wendell (1989) and particularly Barad (2015), I speculate on embodiment, disabilities, assistance and technologies to create a mirrored surface of opportunities allowing for explorations of absence and presence. Such an endeavour presents early design work with the aim to trouble the paternalising paradigm of assistive technologies (Spiel and Gerling, 2021) and identify what the centring of situated and embodied crip perspectives has to offer to a feminist theory of technologies in disabled spaces.

Methodologically sound speculative designs are, according to Auger (2013), ecologically valid if they embed the speculation within a potentially fictional but fundamentally realistic context. I ground my speculations within personal experiences and (quite literally) draw on images of myself to think through the implications of my designs for a theoretical understanding of queer-feminist aspects of disabled technologies. Hence, this work can be understood as creating *verisimilitude* ­– a world that is not actually present but believable – from the position of my personally embodied experiences.

## Transreal tracing: Exploring imagery of presence and absence

Combining theories on gender and disability appears adequate given the gendered dimensions of disability and the disabling dimensions of gender, particularly pertaining to trans existences. This is exemplified vividly in how accessible bathrooms persistently erase gendered identities. More fundamentally, ‘[t]he mannerisms that help define gender […] are all based upon how nondisabled people move. A woman who walks with crutches does not walk like a “woman”’ (Clare, [2009] 2015: 130). Simultaneously, the aforementioned bathrooms are often the only safe option for trans folks – essentially rendering us as symbolically disabled. More parallels can be drawn, though, as ‘the oppression of disabled people is closely linked to the cultured oppression of the body. Disability is not a biological given; like gender, it is socially constructed from biological reality’ (Wendell, 1989: 104). Boundaries drawn along gender are as artificial as those drawn along dis/ability (Goodley, 2014) and these boundaries are fundamentally exertions of power.

The starting point for my speculation is tied to the text ‘TransMaterialities: Trans*/Matter/Realities and Queer Political Imaginings’ by Barad (2015). In the article, Barad argues for an understanding of trans identities as indeterminate, perpetually potential and radically open. By referencing Stryker (2011), a trans theorist reclaiming monstrous identities, the work offers a point of resonance for disability scholars.^6^ I follow their lead in that ‘[l]ike lightning, this article is an exploration of charged yearnings and the sparking of new imaginaries’ (Barad, 2015: 387). The main questions carrying through my speculative explorations are: How can we design for disabled bodies by tracing opportunities rather than rendering them as deficient? How can we design in ways that are grounded in an understanding of disabilities that attends to ‘charged yearnings’ of potentials?

While the specifics of implementation can also be speculated on, here I present only imagery created through the idea of a Transreal Tracing Device. The resulting explorations could be for personal reflection or public display in the contexts of art and provocation. Further potentials for technological use cases lie in creating a social media application where a range of people can explore the relevance of the process for themselves. While inquiries into assistive technologies frame the speculation, the images stemming from Transreal Tracing are intended as speculative thought experiments guided by the design of desired outcomes as opposed to technological specificity.

**Figure 1.**
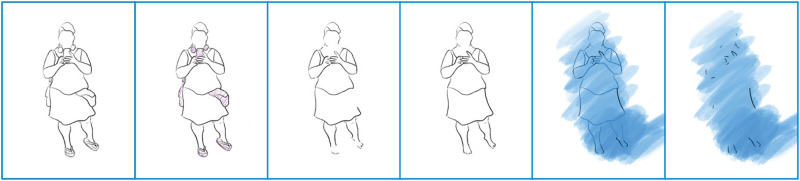
Full body exploration.

**Figure 2.**
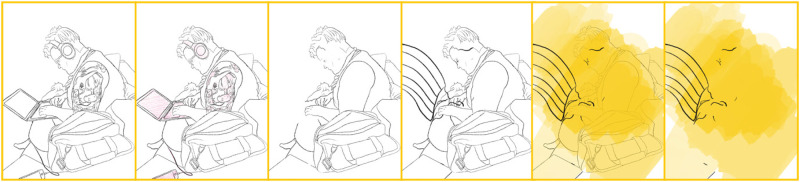
Exploration of a partial body sitting.

**Figure 3.**
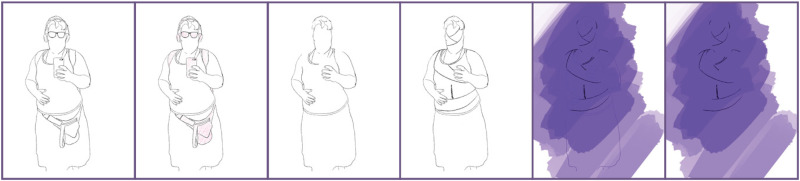
Exploration of a partial body standing.

**Figure 4.**
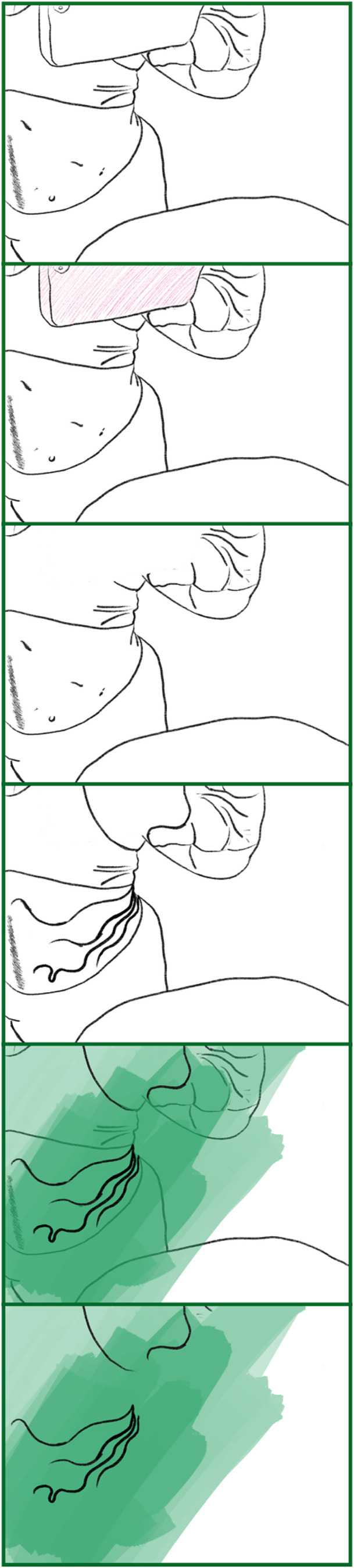
Exploration of the scarred belly.

**Figure 5.**
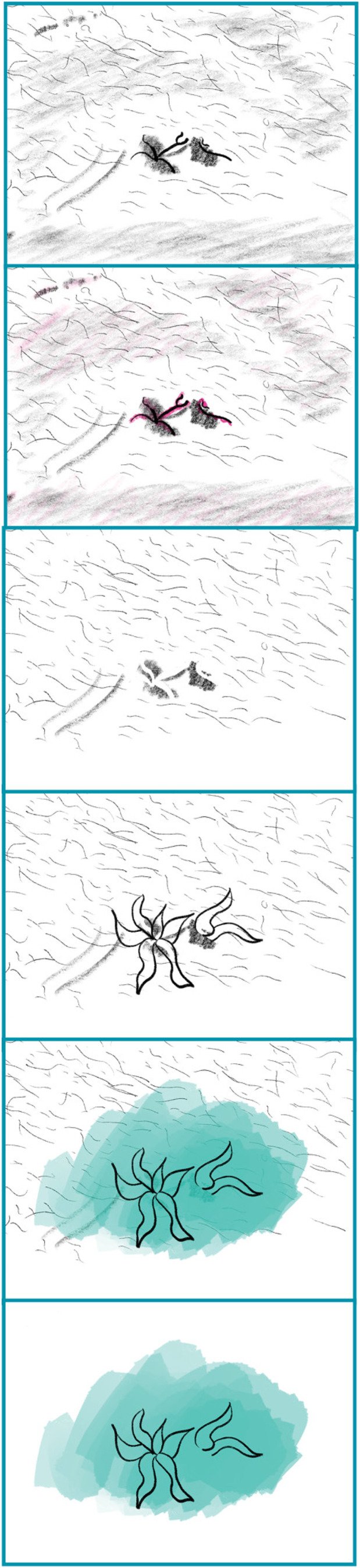
Exploration of a fresh scar.

**Figure 6.**
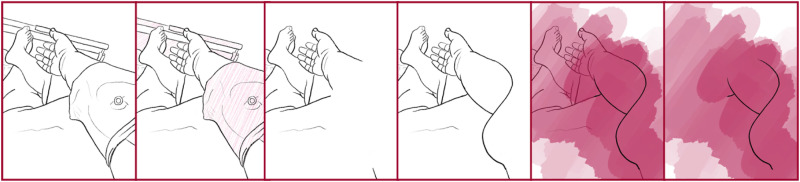
Hospital body exploration.

**Figure 7.**
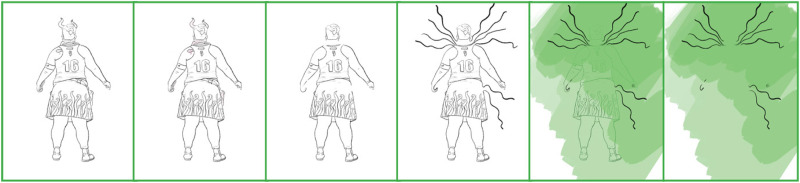
Sports body exploration.

**Figure 8.**
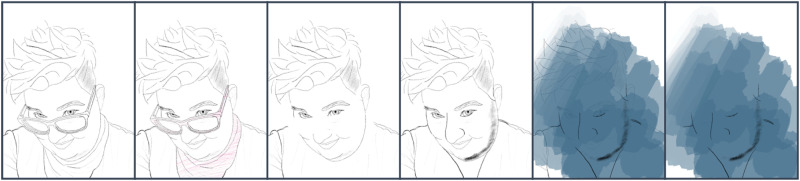
Facial exploration.

In response to this, a Transreal Tracing Device (1) traces disabled bodies, (2) identifies assistance on/around said bodies, (3) removes associated items, (4) refills the blank areas, (5) adds a colour layer to differentiate between what remains from the original drawing and what has been added to the blanks and, finally, (6) removes the initial base, essentially leading to an image of transcendence (see: Figures 1–8). The process identifies how for disabled bodies ‘[o]ntological indeterminacy, a radical openness, an infinity of possibilities, is at the core of mattering’ (Barad, 2015: 401). Figure 1 illustrates Transreal Tracing in action. Assistance can be technological and specific (e.g. the headphones), technological and generalised (e.g. the smartphone) or entirely analogous (e.g. the hipbag holding medicine). What happens in Figure 1 additionally is that in removing the smartphone and inserting the hands, they allow fidgety fingers, coded as disabled, to take a more prominent place. By removing assistance, in this case, we find ourselves confronted with a disabled rawness that has previously been occluded by the use of assistive technologies; it turns out that in tracing the potentials of the image, we recognise the above-mentioned tendency of assistance to occlude disabled ways of being. The Transreal Tracing Device proposes here an artful exploration of disabled embodiments, increasing their visibility and legibility and leading to the personal appreciation thereof.

All illustrations from the speculative Transreal Tracing Device are done on photos depicting (parts of) my body. This is mainly because in creating the initial sets I realised just how intimate this process is, how vulnerable it is to explore one’s disabled embodiments. It seemed inappropriate to take someone else's image and speculate on their desires.^7^

I see parallels in this drawing process with the notion of touching the self as ‘an encounter with the infinite alterity of the self. Matter is an enfolding, an involution, it cannot help touching itself, and in this self-touching it comes in contact with the infinite alterity that it is’ (Barad, 2015: 399). Figure 2 engages with this notion of involuntary touch by showing how the Transreal Tracing Device – without changing the privacy- and seclusion-seeking position of my body – opens up slight potentials, stays with the hands as engaging with an environment but also illustrates an absence beyond the boundaries of the image that remain unaccounted for.

On another note, the image defines ‘on-body’ modifications, in this case an elaborate set of tattoos, as assistive. I had started this specific tattoo after a surgery to regain agency in light of dealing with fresh, involuntary incisions. In working through external pain and trauma by internalising it and letting it free, tattoos can be understood as assistive and supportive and even as ‘desirable’ modification. Through Transreal Tracing, I had the opportunity to let them go, to make space for different potentials. Tattoos to me are a comparatively easy way of regaining agency over not only the integrity of my body after surgery, not only the pain I choose to engage with but also over how my body looks, presents itself, is formed and what it communicates to others.

Stepping further, the Transreal Tracing Device does not just make disability visible by insertion into an emptied space, but also, in stepping through the process, it allows for the identification of altering potential more generally. In following ‘matter's ongoing experimenting with itself—the queer dance of being-time indeterminacy, the imaginative play of presence/absence, here/there, now/then’ (Barad, 2015: 407), Figure 3 makes visible a scar that tends to be occluded. In creating empty space and articulating it as potential, this space functions as a lens for areas previously perceived as fixed. I play with presence and absence by recalling the surgeries and manifesting the scarred inscription they left on my body. Stepping through the Transreal Tracing procedure opened up a space to show the masking that I associated with many pictures of my face; that irritation of not being able to express myself in the ways I want to and, instead, mimicking facial expressions that I painstakingly learned to appropriately emulate.

Said inscription is tied to complex and complicated emotional states. The scars that mark my body are not just markers of fundamentally invasive, painful and repeated procedures; transcending the very notion of a scar can articulate the simultaneously empowering and excruciating aspects tied to mine (see: Figure 4). ‘Feminists have celebrated the body, emphasizing aspects of bodily experience that are sources of pleasure, satisfaction, and feelings of connection. This has led feminists to overlook or underestimate the fact that the body is also a source of frustration, suffering, and even torment’ (Wendell, 1993: 117). However, to some degree, holding that pain, living with it, negotiating being with it, can be a source of pride as well. Through Transreal Tracing, we create images that are open for interpretations, that allow for pain and pride simultaneously, holding a tension that disabled people are often expected to dissolve even though my lived experiences contain shades of each as multitudes. Transreal Tracing might yet dissolve another binary and open options for ambivalence, procedural feelings and, again, indeterminacy.

Barad echoes these multitudes by positioning ‘[r]epulsion at the core of attraction’ (2015: 397). In Figure 5, I drew on an image of a healing scar, an image I find revolting and with which I chose to engage precisely for that reason. The skin around it is angry from repeatedly being connected and disconnected from supposedly protective material. The scar, at this point, is tied to ‘primordial ooze’,^8^ still figuring out how to shape itself, painful, disruptive, progressive. Held together by strings, there are set limitations, but the scar itself was still forming, tracing its potentials. Transreal Tracing here depicts the potentiality of that state; it illustrates pathways. Similar to the bioelectric tracings on the face of the embryonic frog, through Transreal Tracing the scar ‘does not yet exist but only exists in potential for a brief moment and then vanishes’ (Barad, 2015: 405).

In its orientation towards potential and the active act of assistance erasure, Transreal Tracing does not constitute a practice without problems. I alluded above to the notion that we could, following Bennett et al. (2018), value interdependence as core to disabled experiences. We could understand assistive tokens, devices and technologies as a material interdependence, one that with fabrication methods and customisation could in itself be understood as a mode of potentiality. However, I would argue that, in effect, the result is standardised and technocratic. In Figure 6, I explore this within a hospital setting. The needle in my arm together with the gauze put on me within procedures develops into a soft and unreal arm. In this, the removal, insertion and final aesthetic provides a suggestive space indicating a desire for less rigidity. Transreal Tracing is not meant to replace the needle that has a specific medical purpose, but it allows us to imagine an alternative where it seems possible to do things differently while attending to their limitations.

Zooming out again, I attended to a picture that symbolises strength and power. Disabled people are not necessarily conceptualised as strong or powerful. By choosing to show my body in such a situation (see: Figure 7), I engage in a double bind. My body becomes an unlikely disabled body while risking endorsing dominant ableist ideals:

[We] idealize the human body. Our physical ideals change from time to time, but we always have ideals. These ideals are not just about appearance; they are also ideals of strength and energy and proper control of the body. […] Some people can have the illusion of acceptance that comes from believing that their bodies are ‘close enough’ to the ideal, but this illusion only draws them deeper into identifying with the ideal and into the endless task of reconciling the reality with it. Sooner or later they must fail. (Wendell, 1989: 112)

I decided to let the body fail, to embrace the monstrosity that may emerge from it visually. ‘Like the monster, I am too often perceived as less than fully human due to the means of my embodiment’ (Stryker, 2011: 84). I desired my monstrous self to be ‘the spontaneous, brutal, but consequently natural form of the unnatural’ (Foucault, 2003: 56). In that, I used Transreal Tracing as a way of reconciling both my trans identity and my disabled identity, both of which are often rendered unnoticeable (by which I mean systematically ignored), particularly in contexts that operate strictly within an ableist gender binary, such as sports. I speculate on deliberately shedding the ways in which I contribute to my own passing (cf. Samuels, 2013).

My final Transreal Tracing speculation returns to the aspect of trans imaginings more directly in Figure 8. Like Barad’s embryonic frog, I was able to trace potentials and converge on a potential, on an alternative, just to know. Similar to my relationship with my scars, I had to regain agency over my hormone levels as medical staff decided early in my life that these hormones were outside a binary norm and, hence, in need of ‘correction’ and ‘cure’ (Clare, 2017). From this point of deliberate alteration as a default, I had to find a place in which my body could regain access to previously erased potentials. With my rejection of medicalised ‘corrections’ of hormone levels, my face begins and as of the time of writing continues to explore one such potential. It represents ‘a virtual exploration of what might yet be/have been’ (Barad, 2015: 410); and while all speculative sketches have that function, with this one I might be able to explore potentials for bodily modification and alterations with the explicit personal desire to actualise them.

## Desiring disabled technologies

Speculating on transreal images from a disabled perspective means engaging with realities and utopias of lived experiences. Transreal Tracing not only illustrates opportunities for reflecting on paradigmatic stances for assistive technologies more generally, but it also furthers our understanding of the fundamental changes we require to design from perspectives that allow us to negotiate disabilities as markers of ‘rights, not charity’, of pride and tied to an appreciation of disabled bodies (Morris, 2014) as just individual body instantiations (Barnes, 2016) within a broad range of potentials (Barad, 2015).

As described earlier, the design of assistive technologies draws predominantly on a medicalised perspective, one that aims to better, to correct, to fix; but also, one that tends to discipline, eradicate and other (cf. Spiel et al., 2019). Subsequently, disabled people are warily suspicious while acknowledging the necessity of technological support often for our survival as ‘[l]ike cure itself, the evolution of its technologies sparks ambivalence. We fear the shifts. We resist them. We welcome them. We need them’ (Clare, 2017: 94). However, even if technological design operates from a decidedly social model, disabled people and their experiences become secondary considerations framed as nuisances, associated with increased costs and only useful if they do not disrupt the status quo:

The spaces that are provided or modified in some way […] remain distinctly provisional spaces, in which disabled people are ‘provisionally’ allowed so long as they seek to inhabit, utilise and conduct themselves in these spaces as would a non-disabled person. […] [M]any disabled people once again feel ‘out of place’, being left to explain or even to justify their presence under the most mundane of circumstances. (Hansen and Philo, 2007: 499f.)

Hence, neither a mainly medical nor a singularly social model are suitable as exclusive perspectives from which to engage in the development of assistive technologies that positively negotiate disabilities from a self-determined^9^ position. To allow ourselves and our environments to appreciate disabled lives, to design from within disabled experiences, we need to find ways of creating technologies facilitating positively connoted conversations about disabilities. We need to create technologies that are fundamentally reoriented towards celebrating and supporting *life* with disability as opposed to eradicating and removing its visible effects. This is not to deny that disabled bodies cannot be experienced as painful, restricting or hindering – they often are (Wendell, 1993) – rather, designers should shift their/our^10^ focus. As it stands, they/we often implicitly and inherently foster the power structures that non-disabled people exert over disabled people by identifying assistive requirements from medical literature and the social sciences. Instead, they/we urgently need to reshift their/our focus towards decidedly and explicitly attending to disabled people with our/their identified desires and needs, to acknowledge the transformative characteristics of our/their designs – one way or another. ‘We must ground our work in a foundation of humility. We must be humble enough to recognize that none of us are the “modest witness” of “objective, impartial, pure” scientific authority’ (Williams and Gilbert, 2019).

Participatory Design can comprise one way to do so. However, it systematically risks tyrannising disabled people into contexts that remain driven by ableist external research interests and design agendas.^11^ I encourage designers to decidedly *love* disability cultures within a frame of loving epistemology (cf. De Jaegher, 2019). Transreal Tracing Devices could function as a token of self-love. ‘Disabled people can participate in marginalizing ourselves. We can wish for bodies we do not have, with frustration, shame, self-hatred. We can feel trapped in the negative body; it is our internalized oppression to feel this’ (Wendell, 1989: 113). Transreal Tracing then could offer a counterpoint, one that encourages decided engagement with the disabled and/or trans self, and shift the internal focus towards an appreciation of artful explorations fundamentally tied to bodily experiences. It can be an answer to Wendell's early critique: ‘[F]eminist theory has not taken account of a very strong reason for wanting to transcend the body’ (1993: 118). It does so by transcending the modes that are tethered to the physicality of disabled bodies and creating situated contexts for appreciation.

The exploration along notions of Transreal Tracing offers an opportunity to reconfigure how designers and researchers understand technological concepts around disabilities. Instead of centring assistance, they/we could *structurally* centre disabled perspectives as desirable. Instead of asking how to mitigate and remove the intricacies of disabled experiences, they/we could embrace us/them and support dismantling the hostile environments we/they live in with enthusiasm for difference. For this, designers will need to immerse them/ourselves into disability cultures they/we are designing with. They/we need to dis-able the paternalistic discourses surrounding assistive technologies and design these technologies not just around orientations towards ‘functioning’, but most of all they/we need to start desiring *disabled and disabling technologies* as a proud, not prejudiced (Morris, 2014), stance. They/we can reconfigure what technologies should be able to do, how they can be non-assistive but still specific and helpful, how they can be appropriated, how they can provoke and how they can embody appreciation and kinship, value interdependence and support access intimacy, as:

[a]ccess for the sake of access is not necessarily libratory, but access for the sake of connection, justice, community, love and liberation is. We can use access as a tool to transform the broader conditions we live in, to transform the conditions that created that inaccessibility in the first place. Access can be a tool to challenge ableism, ablebodied supremacy, independence and exclusion. (Mingus, 2017)

The concept of Transreal Tracing might offer a contribution to access intimacies as a form of interdependence and feminist theories around disabilities in illustrating how we might talk about potentials, about transcendence and transformation, about agency and choice and about desire. It further strengthens the parallels and connections between queer and disabled lives. It can aid to a ‘potent political formation’, one that forms ‘possibilities for alliance with nature's ongoing radical deconstruction of naturalness’ (Barad, 2015: 413). As it stands, using speculations through the Transreal Tracing Device allowed me to explore my initial hunch that the potential that Barad sees for trans identities and the charged yearning of monstrous matter in this context can be productive for disabled identities as well. It allows us to understand, conceptualise and appreciate disabilities not as deficit or ‘mere difference’ (Barnes, 2016), but rather as curious and charged potentials with opportunities for exploration, design, reflection and art. Hence, the theoretical contribution offered through the speculations lies in materialising this potential through sketched illustrations that question the visual representation of disabilities but even more so provide alternative modes for thinking through disabled identities and assistive technologies as productive potentials instead of fixed and rigid problems.

Even beyond this theoretical contribution as a thought artefact (akin to ‘thought experiments’ in Cognitive Sciences), Transreal Tracing offers more tangible implications for how we might think about ‘assistive’ technologies in theory and practice, research and design. With Figures 2 and 8, there is an indication that assistive technologies could – instead of deriving themselves from a notion of fixture and cure (Clare, 2017) – understand their/our field of action as one opening up potentials for the *desires and joys*, the yearning and wishes that disabled people (and particularly queer/trans disabled people) have for our/their technologies. These might be similar or radically different from the interests of non-disabled people; without actors in the field of assistive technologies reframing what they/we mean by assistance as something that supports self-determination (Williams, 2018a), they/we have no structural insights to offer. However, for me personally, Transreal Tracing shows how regaining the agency over how my body transforms along lines of medical intervention (Figures 4 and 5) as well as gendered perception (Figure 8) can have supportive (or ‘assistive’) effects even in cases where the potential traced is not one likely to actualise. Such a move would additionally require us to work against normative tendencies in computing (cf. Spiel, 2021), and celebrate and curiously explore bodily differences, unique aspects and specificity as I intended to do in Figure 1. This comes with acknowledging strength in nuanced ways (see: Figure 7), seeing it in moments when pain becomes unbearable and is yet held (see: Figure 5), moments that can be a point of aesthetic engagement in their own right, as explored in Figures 3 and 6. Hence, Transreal Tracing shows how assistive technologies can be understood as potentials that *curiously explore self-determined desires and joys to develop aesthetic supportive devices that acknowledge nuanced dimensions of strength*.

All images show subtle traces of aspects hinting more or less prominently to negotiations of gendered identities. This understanding and redefinition of assistive technologies as disabled technologies­ (in self-determined support of disabled people) does require a stepping away from normative assumptions of curing and mitigating disability. But it also requires an awareness of gendered dimensions, particularly in how they are hoisted onto disabled people either as ignoring individual sexual desires and identities or as overriding queer identities with cis-heteronormative assumptions. In that regard, this redefinition above has its own potential to apply to technologies in marginalised contexts more generally.

## Conclusion

Taking the notion of assistive technologies as a starting point, I set out to trouble the concept by (literally) drawing on Queer Theory and Critical Disability Studies. Given the systematic exclusion of disabled people in dominant discourses around expertise, technologies, needs and design, we/they remain structurally isolated from solidaric peer networks that celebrate crippled cultures (see also: Morris, 2014). Using speculative design as a methodological approach to reflect on desirable utopias, I visualised a series for transformations that allow us to think further on the notion of what we understand as assistance, how the same procedures can render disabilities visible and invisible. The resulting images make an argument for re-thinking assistance with the potential to embody personal and political support; to create imagery for the self with the intent to bolster confidence in demanding alternative political action and, ultimately, self-determination in representation as well as technological research and design. In that, I used *technology* as a rhetorical token, as a playing field for inspiration rather than engaging with specifics on a system level. I argue that doing so allows us to escape the medico-technical determinism that plagues the field of assistive technologies as it stands.

Engaging assistance and technologies in such a way does not just apply broader ranges of feminist thinking into a specific context of marginalisation; doing so mirrors back into feminist theories as well. In conducting my work, I drew out parallels between trans and disabled experiences around the potentials of imaginings and desire, fantasy and projected materiality. Being trans and disabled, I found myself exploring the differing and shared aspects of my identities: living with a permanent mismatch between how my body is identified vs how I live with it; how this body is conceptualised as deficient vs a nuanced understanding of the support and strength it provides me with; a body rendered undesirable in multiple ways vs a body that loves and desires; a body that does not match up to expectations and assumptions. However, my work so far is a starting point for explorations of how we can think on the potentials of disabilities through an understanding of trans experiences.

With the status of such a starting point, the work is necessarily incomplete. My body is a white body, one that has only ever lived within western contexts. Hence, there is potential in understanding this work with additional lenses from Critical Race or Postcolonialist Theories. My disability operates at the uncanny valley of passability, a privilege that allows me to put efforts into assuming a non-disabled identity strategically as a matter of safety or protection in a fundamentally ableist world (cf. Woolwine and Dadlez, 2016).^12^ Additionally, I draw from a perspective as a trans and disabled scholar; hence, how this approach might relate to cis disabled people remains an open question. Some readers might also be frustrated about the lack of concrete technological guidance in my work. We will yet have to find out what it means to tie this speculative theoretical exploration back into a more physically material(ised) world – and I am excited about the potential.

## Notes

1. I use identity-first language when referring to a group of disabled people to avoid artificially constructing language around disability (see: Sinclair, 2013; Andrews et al., 2019). However, personal preferences differ between individuals and should be respected as such.

2. Together with Mingus (2017), ‘I understand Access Intimacy as something that can transform ordinary access into a tool for liberation, instead of merely reinforcing “inclusion” and “equality”’.

3. Edelman (1974) discusses the ideologies and power structures deeply entrenched in the language of ‘the helping professions’ in depth.

4. In quite the opposite way to how HCI Games research predominantly conceptualises games for neurodivergent individuals (cf. Spiel and Gerling, 2021).

5. Note that internalised ableism exists for disabled people as well and if not consciously challenged might lead to the same kinds of technologies (cf. Low, 2006).

6. I am thinking here of the multi-facetted conceptualisations of the term ‘freak’ as discussed, for example, in Garland-Thomson (1996).

7. This would also be fundamentally irresponsible, at least in the explorative stages that this project is in at the time of writing, since ‘[i]n an important sense, in a breathtakingly intimate sense, touching, sensing, is what matter does, or rather, what matter is: matter is condensations of responses, of response-ability’ (Barad, 2015: 401). However, in participatory conversations and by establishing access intimacies, this process might have potential as a collective one.

8. Barad uses the term in a list of charged potentials. ‘Electrical energy runs through disparate topics in what follows: lightning, primordial ooze, frogs, Frankenstein, trans rage, queer self-birthing, the quantum vacuum, virtual particles, queer touching, bioelectricity, Franken-frogs, monstrous re/generations’ (Barad, 2015: 388).

9. The use of the term ‘self-determination’ here implicitly references and inverts the other-determination that disabled people, particularly autistic individuals, are all too often subjected to (cf. Williams, 2018a).

10. I start using the phrasing ‘they/we’ here (in the context of design) and ‘we/they’ (in the context of disability) to position myself as a disabled designer within this entangled mess.

11. Cook and Kotari (2001) have expanded more on the notion of how participatory approaches can create tyranny.

12. Though, passing privileges are fundamentally granted. I can put increased efforts into trying to pass but they are limited by the implicit willingness of my environment to grant me plausible deniability (see also: Williams, 2018b).
